# Ensemble Tractography

**DOI:** 10.1371/journal.pcbi.1004692

**Published:** 2016-02-04

**Authors:** Hiromasa Takemura, Cesar F. Caiafa, Brian A. Wandell, Franco Pestilli

**Affiliations:** 1 Center for Information and Neural Networks (CiNet), National Institute of Information and Communications Technology, and Osaka University, Suita, Japan; 2 The Japan Society for the Promotion of Science, Tokyo, Japan; 3 Graduate School of Frontier Biosciences, Osaka University, Suita, Japan; 4 Department of Psychology, Stanford University, Stanford, California, United States of America; 5 Instituto Argentino de Radioastronomía (IAR)—CCT La Plata—CONICET, Villa Elisa, Buenos Aires, Argentina; 6 Department of Psychological and Brain Sciences, Indiana University, Bloomington, Indiana, United States of America; 7 Programs in Neuroscience and Cognitive Science, Indiana University Network Science Institute, Indiana University, Bloomington, Indiana, United States of America; Oxford University, UNITED KINGDOM

## Abstract

Tractography uses diffusion MRI to estimate the trajectory and cortical projection zones of white matter fascicles in the living human brain. There are many different tractography algorithms and each requires the user to set several parameters, such as curvature threshold. Choosing a single algorithm with specific parameters poses two challenges. First, different algorithms and parameter values produce different results. Second, the optimal choice of algorithm and parameter value may differ between different white matter regions or different fascicles, subjects, and acquisition parameters. We propose using ensemble methods to reduce algorithm and parameter dependencies. To do so we separate the processes of fascicle generation and evaluation. Specifically, we analyze the value of creating optimized connectomes by systematically combining candidate streamlines from an ensemble of algorithms (deterministic and probabilistic) and systematically varying parameters (curvature and stopping criterion). The ensemble approach leads to optimized connectomes that provide better cross-validated prediction error of the diffusion MRI data than optimized connectomes generated using a single-algorithm or parameter set. Furthermore, the ensemble approach produces connectomes that contain both short- and long-range fascicles, whereas single-parameter connectomes are biased towards one or the other. In summary, a systematic ensemble tractography approach can produce connectomes that are superior to standard single parameter estimates both for predicting the diffusion measurements and estimating white matter fascicles.

## Author Summary

Diffusion MRI and tractography opened a new avenue for studying white matter fascicles and their tissue properties in the living human brain. There are many different tractography methods, and each requires the user to set several parameters. A limitation of tractography is that the results depend on the selection of algorithms and parameters. Here, we analyze an ensemble method, Ensemble Tractography (ET), that reduces the effect of algorithm and parameter selection. ET creates a large set of candidate streamlines using an ensemble of algorithms and parameter values and then selects the streamlines with strong support from the data using a global fascicle evaluation method. Compared to single parameter connectomes, ET connectomes predict diffusion MRI signals better and cover a wider range of white matter volume. Importantly, ET connectomes include both short- and long-association fascicles, which are not typically found together in single-parameter connectomes.

“This is a *PLOS Computational Biology* Methods paper”

## Introduction

Tractography uses diffusion-weighted magnetic resonance imaging (diffusion MRI) data to identify specific white matter fascicles as well as the connections these fascicles make between cortical regions [1–6]. Specifying the pattern of connections between brain regions (“connectome”) is a fundamental goal of neuroscience [7–9]. One of the major goals of tractography is to establish a model of the complete collections of white matter tracts and connections (“structural connectome”, also referred as “tractogram”) in the human brain. Hereafter, we refer to structural connectomes estimated using tractography as “connectomes” or “connectome models”.

A variety of tractography algorithms are in wide use [10–18](see “Related literature” in Discussion). These algorithms calculate streamlines (also called “estimated fascicles”) through the white matter using somewhat different principles. Some tractography methods (local tractography) calculate streamlines by tracking the orientation of diffusion signal locally and step-wise based on deterministic [10,19–21] or probabilistic selection methods [11,12]. Other tractography methods (global tractography) reconstruct the trajectory of streamlines based on goodness-of-fit to diffusion signals [16,22–31]. Each algorithm offers some advantages and disadvantages.

For any tractography method, investigators must set parameter values. Key tractography parameters include maximum and minimum streamline length, seed selection, and stopping criteria for terminating a streamline, and the minimum radius of curvature allowed for building each streamline. Differences in parameter values yield differences in streamlines [32–39]. The parameter dependency of tractography has been observed in both local and global tractography algorithms [34].

**Fig 1:**
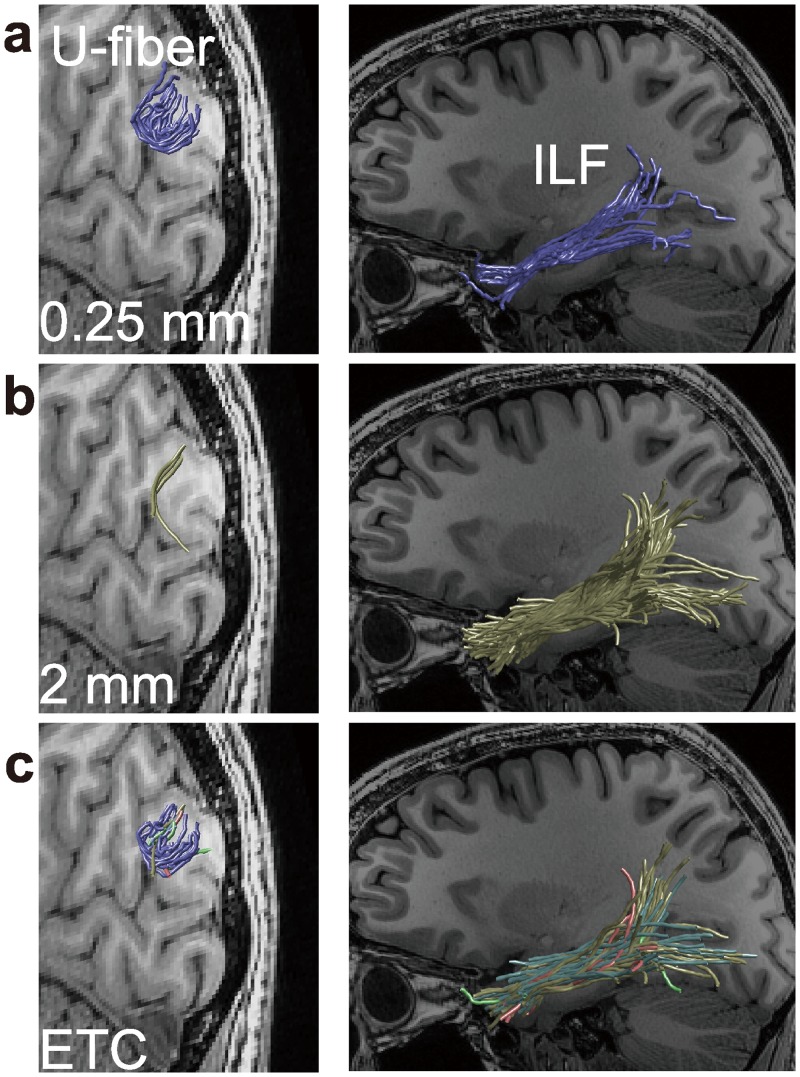
Short- and long-range fascicles supported by different parameter selections. The two columns compare short-range fascicles (left, U-fiber) connecting V3A/B and V3d and long-range fascicles (right, the inferior longitudinal fasciculus; ILF) segmented from different connectome models. The images show extremely different estimates using a low minimum radius of curvature threshold (**a**, 0.25 mm) and high threshold (**b**, 2 mm). **a.** The 0.25 mm results show a dense set of short-range fascicles, but a thin set of long-range fascicles. **b.** Conversely the 2 mm results show sparse short-range fascicles and dense long-range fascicles. **c.** Ensemble Tractography generates connectomes including both short- and long-range fascicles. Streamline colors in **c** indicate different parameter settings used to generate the streamlines (blue, 0.25 mm; green, 0.5 mm; red, 1 mm; yellow, 2 mm; light blue, 4 mm). Results are shown from one left hemisphere (subject 1, STN96 data set; see Material and Methods).

In common practice, investigators choose an algorithm and set fixed parameter values in the hope of optimizing streamlines for general use. However, recent studies [40,41] demonstrated that no algorithm or parameter values are optimal across all conditions. Specifically, Chamberland and colleagues [41] show that the best choice depends on a variety of factors such as the specific region of white matter or the specific tract being studied. For example, Fig 1 compares two tracts and shows how the best parameter value differs. Tracts between nearby regions on the cortical surface have short association fibers with relatively high curvature (U-fiber; left panels in Fig 1). To identify U-fibers investigators must set parameters that allow tracts with high curvature (top panels in Fig 1). In contrast, the major fascicles of the brain, such as the Inferior Longitudinal Fasciculus (ILF) or the Superior Longitudinal Fasciculus (SLF), have relatively long and straight cores. Better estimates of the core of these tracts are obtained by sampling streamlines with relatively low curvature (middle panels in Fig 1). Additional factors affecting the optimal parameter choice for streamline generation may include diffusion MRI acquisition parameters (e.g., b-value, voxel size and angular resolution). In general, no single parameter value may capture the full range of streamlines globally in every brain.

In the machine learning and statistical classification literature, it has been shown that for large and heterogeneous data sets combining multiple types of classifiers improves performance over single classifier methods (Ensemble methods [42–44], see [45] for a review). The human white matter provides similar challenges, because it contains large sets of heterogeneous fascicles different in length, volume and curvature. Given the complexity of human white matter, ensemble methods incorporating a range of tractography algorithms and parameters may be a valuable approach for improving tractography performance. The idea of incorporating tracts from multiple sources in the initial construction of a connectome has been suggested in earlier publications [27,31].

**Fig 2:**
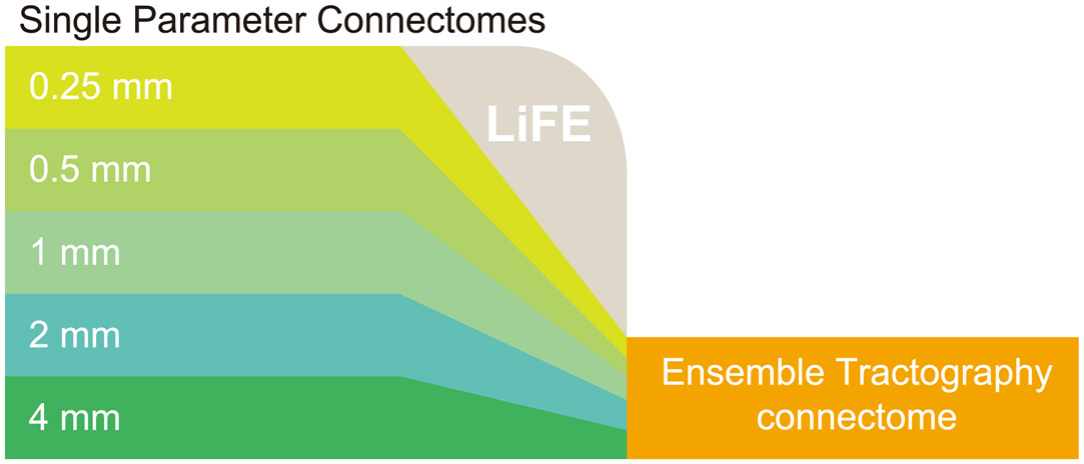
Example of Ensemble Tractography architecture. Using five different curvature thresholds (0.25 to 4 mm), we generated five candidate Single Parameter Connectomes (SPC; green colors). We first combined SPC candidate connectomes to generate a candidate Ensemble Tractography Connectome (ETC). We then generated an optimized ETC using LiFE (see Material and Methods section for technical detail). We also describe alternative ET architecture (see S10 Fig and S1 Text, Section 5).

We describe an ensemble method, which we call Ensemble Tractography (ET), to reduce problems arising from single algorithm and parameter selection. We illustrate the method with an example that addresses the parameter selection problem. First, we create a set of connectomes, each generated using a different parameter setting. These are called single parameter connectomes (SPCs). We then combine streamlines from multiple SPCs into a new candidate connectome, and we use Linear Fascicle Evaluation (LiFE [46]) to optimize this connectome and eliminate redundant streamlines. We call the result the Ensemble Tractography Connectome (ETC). Fig 2 shows a flow diagram of the ET algorithm.

We report two key findings. ETCs (1) include streamlines that span a wider range of curvatures as compared to any of the SPCs, including both short- and long-range fibers (bottom panel in Fig 1), and (2) ETCs predict the diffusion signal more accurately than any SPC.

To support reproducible research, the algorithm implementation and example data sets are made available at an open website (http://purl.stanford.edu/qw092zb0881).

## Results

We evaluated ET with respect to one key parameter the streamline curvature threshold. Here we describe an example ET architecture, and in S1 Text (Section 5), we discuss alternative architectures.

Fig 2 describes the schematic flowchart of the example ET architecture. We analyzed ET using diffusion data from 10 hemispheres. In each hemisphere, we generated five candidate SPCs (minimum radius of curvatures = 0.25, 0.5, 1, 2 and 4 mm [18]). Each SPC candidate was composed of 160,000 streamlines. We combined SPC streamlines to create a candidate ensemble connectome. Finally, we used LiFE to optimize the candidate ETC. Below we compare the properties of each of the five optimized SPCs with the optimized ETC.

### The ETC includes streamlines from multiple SPCs

We now return to the example in Fig 1. All connectomes in Fig 1 were optimized using LiFE. The left-panels show U-fibers connecting two adjacent cortical regions, V3A/B and V3d (see Materials and Methods and S1 Fig). The SPCs with high (1/0.25 mm) and low (1/2 mm) curvature parameters return very different results. The high curvature parameter SPC includes many streamlines, and the low curvature SPC has very few streamlines. The right-panels show estimates of the relatively long-range projections that make up the inferior longitudinal fasciculus (ILF). In this case the situation is reversed: the high curvature SPC has many fewer streamlines than the low curvature SPC. Moreover, the terminations of these streamlines do not show the same branching pattern and do not extend into the occipital lobe.

The images in the bottom panels of Fig 1 show the streamlines in the optimized ETC. The ETC model includes many U-fiber streamlines, similar to the 0.25 mm SPC. The estimated ILF contains the same branching pattern that extends into the occipital lobe as the 2 mm SPC. The color of the individual ETC streamlines indicates its SPC origin. The ETC estimates of the U-fibers include streamlines mainly from SPC that permit high curvature (0.25 mm). The optimized ILF includes streamlines mainly from SPCs with lower curvature (1 to 4 mm). The ETC includes streamlines from all of the SPCs.

### The curvature parameter is not only a bound

Nominally, the curvature parameter is a bound—one should not have higher curvature than the specified level [18]. In practice, however, we find that the bound impacts many properties of the candidate connectome.

**Fig 3:**
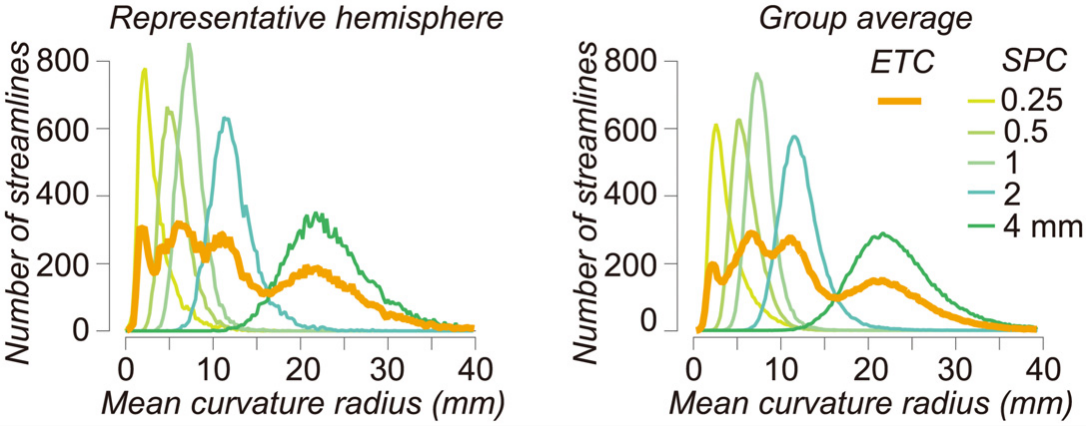
ETC supports streamlines with a wide range of curvatures. Distributions of radius of curvature in optimized connectomes in six connectome models are shown. The results in occipital cortex in one hemisphere (left panel) and group average (right panel, *N* = 10 hemispheres) from STN96 dataset are depicted (see S2 Fig for occipital white matter regions used for analysis in these subjects). Vertical axis is the number of streamlines. Horizontal axis is the mean radius of curvature averaged along individual streamlines. Distributions of the candidate connectomes are shown in S3 Fig. The distributions obtained using the PICo algorithm in the Camino package are shown in S4 Fig.

We illustrate the effect of the curvature threshold on each SPC in the occipital white matter of the 10 hemispheres in STN96 dataset (Fig 3; see Materials and Methods; S2 Fig depicts white matter regions used for the analysis). For each of the bounds we tested, the candidate and optimized connectome curvatures form compact, single-peaked distributions; the peak increases monotonically as the minimum radius of curvature increases (see S3 Fig for distribution in candidate connectomes). When the curvature bound is high (small radius of curvature), the candidate connectome streamlines tend to have a relatively high mean curvature. When the curvature bound is low (high radius of curvature), the candidate connectome tends to have a relatively low mean curvature.

Thus, the curvature parameter is not simply a threshold; it influences the distribution of streamline curvatures in the optimized and candidate connectomes. For this reason, setting a lenient bound on the curvature (i.e., a low value of the minimum radius of curvature) does not yield a good representation of long-straight fascicles (Fig 1). Conversely, setting a strict bound on the curvature (i.e., a high value of minimum radius of curvature) eliminates U-fibers from the candidate connectome. We confirmed that the lenient bound on the curvature does not produce many straight streamlines using other tractography algorithm implemented in a different software package (PICo [11]; S4 Fig, S1 Text, Section 1).

To reduce the curvature bias present in each SPC, the candidate connectome for the ETC combines samples from multiple SPCs whose parameters span a significant curvature range (thick orange line; Fig 3). Hence, the ETC strategy is effective in the sense that ETCs include streamlines with a broader range of curvatures.

### ETC streamline count, density and white matter coverage

**Fig 4:**
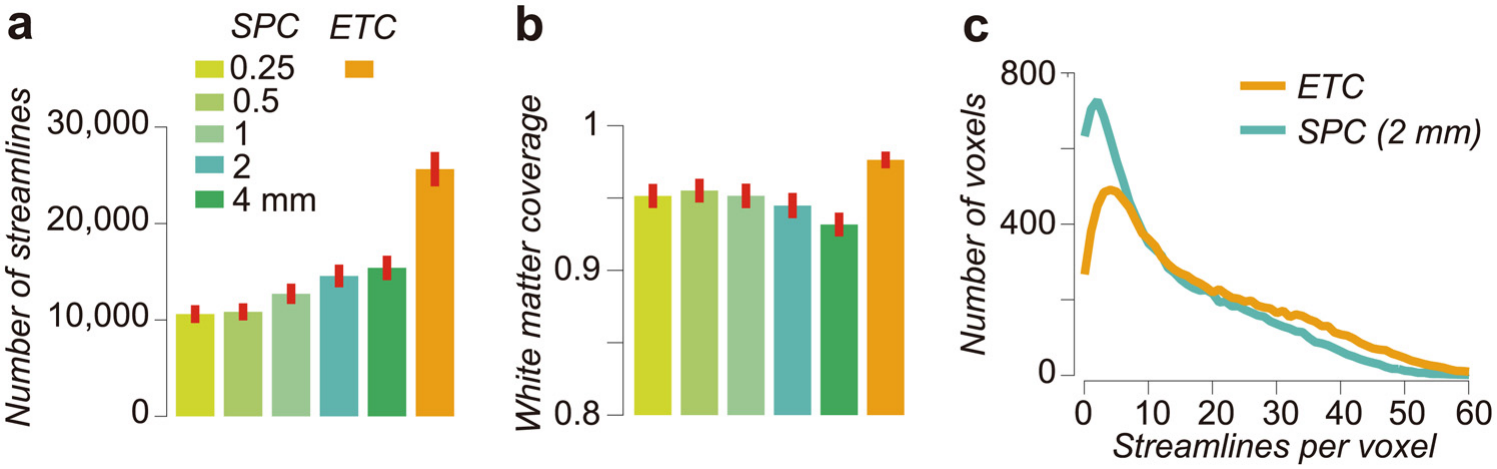
Properties of the Ensemble Tractogrpahy Conectome. **a.** Number of streamlines supported by each optimized connectome model (optimized connectome size). **b.** Proportion of white matter voxels covered by each connectome model (white matter coverage; see Materials and Methods for seeding methods in tractography). Error bars are ±1 s.e.m. across hemispheres. **c.** Streamline density (number of streamline per voxel) in two connectome models (SPC 2 mm and ETC). Vertical axis depicts the number of voxels averaged across 10 hemispheres.

The optimized ETC includes more streamlines than any of the optimized SPCs (Fig 4a). Importantly, nearly twice as many streamlines from the candidate ETC survive the LiFE process and contribute to the diffusion signal predictions.

Typically streamlines generated using whole brain tractography do not pass through all of the voxels in the white matter. For very simple algorithms, such as deterministic tracking based on diffusion tensors [10], as many as 17% of the white matter voxels contain no streamlines (see S8c Fig). We show that ETC streamlines pass through a larger percentage of white matter voxels than any of the individual SPCs (Fig 4b). The streamlines in SPCs (based on CSD and probabilistic tractography methods [18]) cover up to 95% of the white matter, whereas streamlines in the ETC cover up to 98% of the white matter. Because in reality the entire white matter volume contains streamlines, this result suggests that ET recovers more information from the diffusion data. The failure to find streamlines in about 2% of the voxels shows that we continue to miss some fascicles.

While the number of ETC streamlines is nearly twice the number in any SPC, the white matter coverage is only about 3 percent greater. It follows that the number of streamlines per white matter voxel in the ETC is larger than the number in any of the SPCs. Whereas the mean number of streamlines per voxel in the SPCs is around 13, the mean in the ETC is nearly 18. Fig 4c shows a histogram that counts the number of streamlines in each voxel, comparing the 2 mm SPC and the ETC. Notice that many of the voxels (77.9% voxels on average) have more streamlines in the ETC.

The larger number of streamlines within each voxel implies that the ETC streamlines can predict more complex diffusion orientation distribution functions. S5 Fig describes the example crossing fascicle voxel in which ETC predicts diffusion signal significantly better than SPC. This is because each streamline can point in a slightly different direction and thus potentially predict diffusion in more directions. Coupled with the greater coverage across white matter voxels, the ETC should be able to provide a better prediction of the diffusion signal.

### ETC connectome accuracy

**Fig 5:**
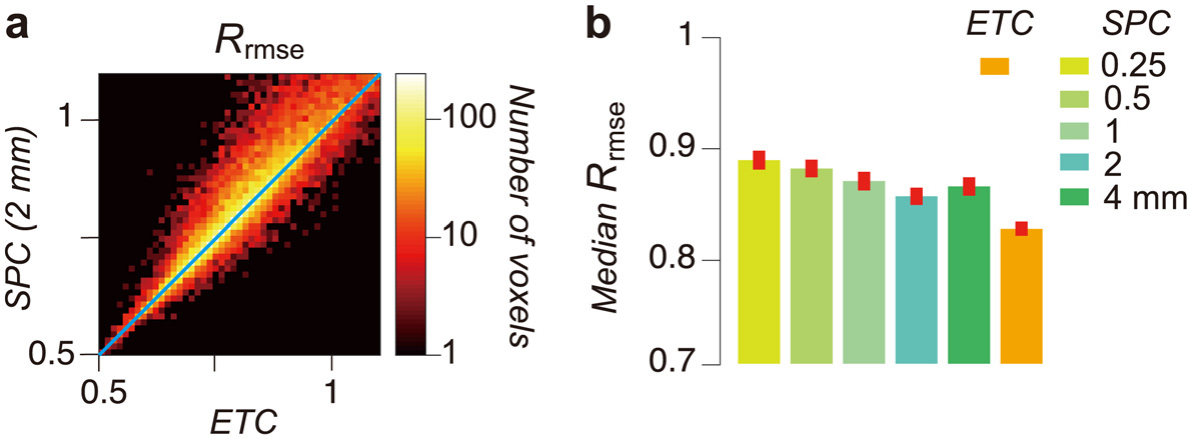
Comparison of SPC and ETC connectome relative error. **a.** Comparison of *R*_*rmse*_ across two representative connectome models (horizontal axis: ETC, vertical axis: SPC 2 mm) in subject 1, left occipital cortex in STN96 dataset. Color chart depicts the number of voxels. In many voxels, ETC error is lower than the error of the 2 mm SPC model. **b.** Comparison of *R*_*rmse*_ across all models in occipital cortex of 10 hemispheres in STN96 dataset. Vertical axis indicates a median of *R*_*rmse*_ across occipital cortex voxels. Error bar depicts ±1 s.e.m. across hemispheres.

Next, we compare SPC and ETC connectome accuracy (Fig 5). Accuracy is evaluated as the ratio of root mean square error between model and data to the test-retest reliability (*R*_*rmse*_ [46–48]; see Eq 3 in Materials and Methods).

Fig 5a shows a two-dimensional histogram comparing the accuracy of the ETC and the 2 mm SPC in a single, typical subject. For large portions of the white matter (62.4% voxels in Fig 5a), accuracy is higher (*R*_*rmse*_ lower) for the ETC than the SPC. Fig 5b describes the median *R*_*rmse*_ of the 6 connectome models (SPC; 0.25, 0.5, 1, 2 and 4 mm and ETC) across all ten occipital lobes. The median ETC accuracy is significantly higher than any of the SPCs.

S6 Fig compares the prediction accuracy of ETC and SPC (2 mm) and tests whether increasing the size of the candidate SPC reduces the primacy of the ETC over the SPC (see S1 Text, Section 2). In this comparison, we matched the size of candidate SPC to that of ETC (800,000 streamlines; BigSPC model; see S1 Text, Section 2). The optimized BigSPC supports as many streamlines as the ETC (S6b Fig) but the ETC covers a larger portion of the total white matter volume (S6c Fig). Importantly, the prediction accuracy of ETC is consistently higher than BigSPC (S6d Fig; see S6e Fig for comparison in individual hemispheres).

### The optimal parameters vary between white matter pathways

**Fig 6:**
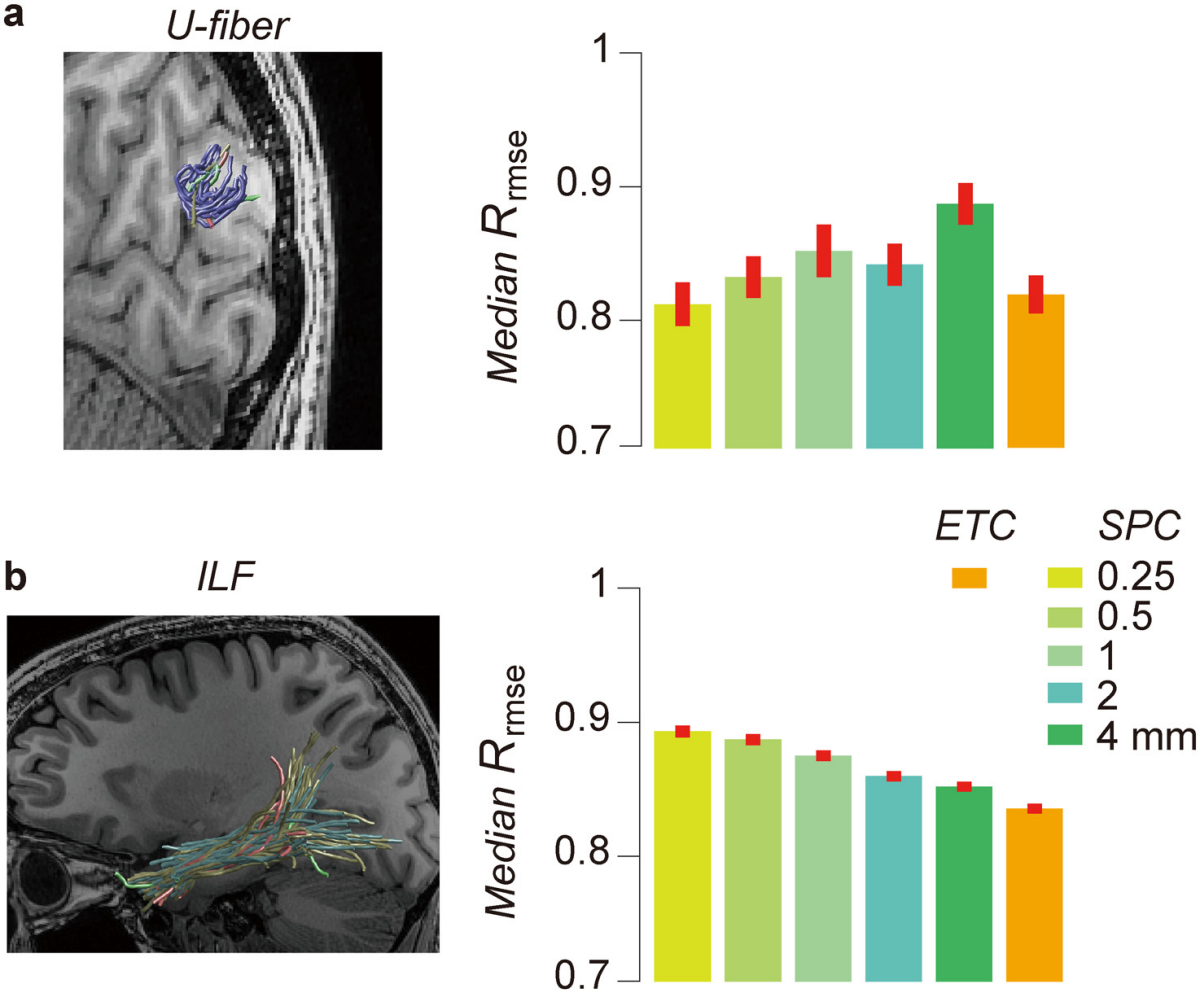
Changes in optimal parameter between different white matter regions. Comparison of *R*_*rmse*_ in voxels along white matter pathways (**a.** U-fiber; **b.** ILF; see Fig 1) across connectome models. Within SPC models, 0.25 mm performs slightly better along U-fiber whereas 4 mm performs slightly better along the ILF. ETC performs better in both pathways. Error bar depicts ±1 s.e.m. across voxels.

Fig 6 compares connectome model accuracy between different white matter pathways (U-fiber and the ILF, as shown in Fig 1). We compared the accuracy of six connectome models in the voxels defined by the best U-fiber (Fig 6a, left, ETC U-fiber) and ILF (Fig 6b, left, ETC ILF) within the same hemisphere of the same subject. In all SPC models, 0.25 mm curvature threshold produces the best performance as compared with other thresholds in the U-fiber voxels, whereas the 4 mm SPC performs better than others in the ILF voxels (Fig 6b). This shows that the best SPC differs between white matter pathways and brain volumes. In both U-fiber and the ILF, ETC model performs similarly or better than the best SPC model (Fig 6).

### ETC performance evaluated in the total white matter volume

**Fig 7:**
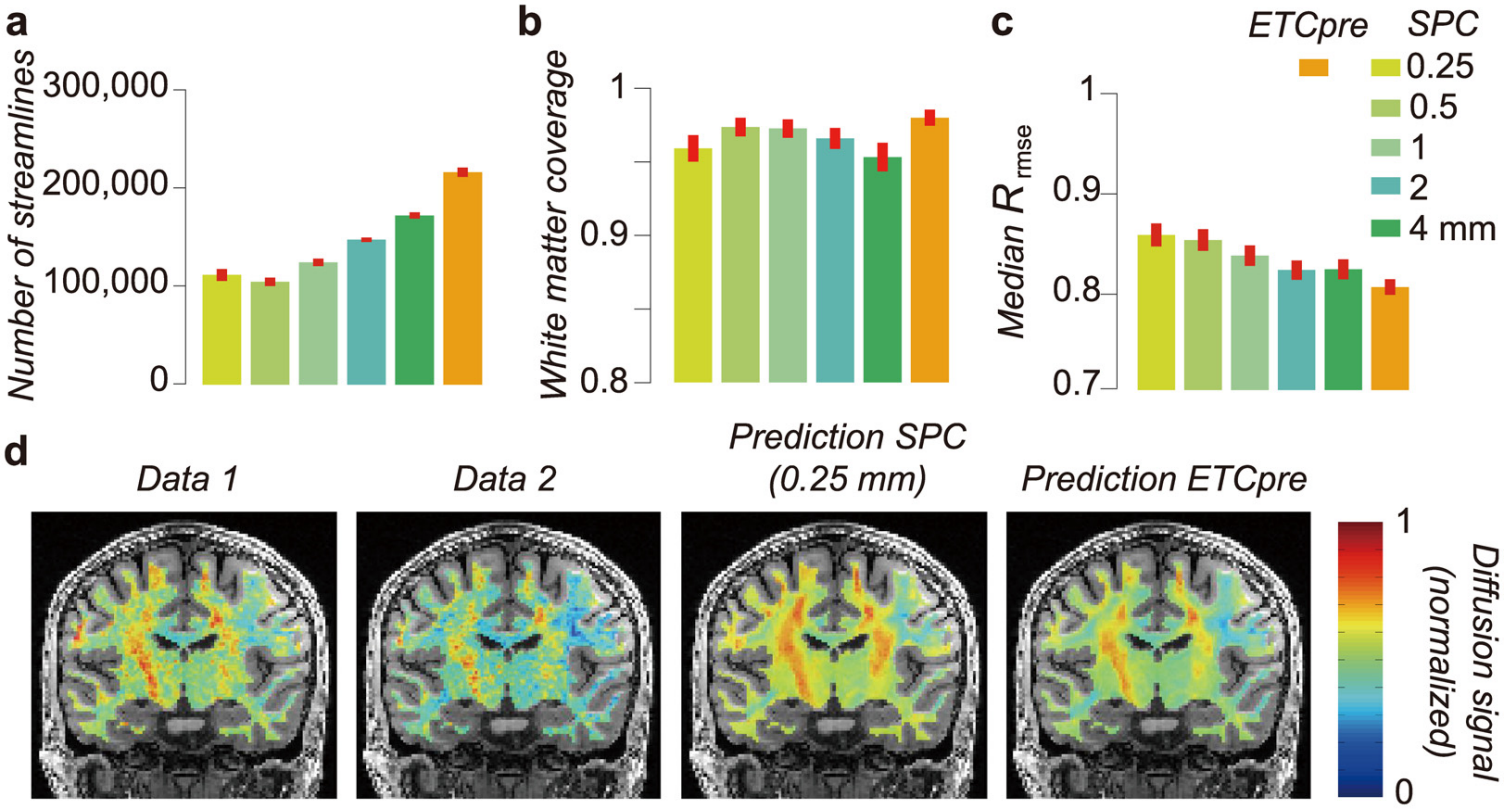
Whole brain ETC performance. **a.** Optimized connectome size of SPCs and ETC with preselection (ETCpre; see S1 Text, Section 5) using whole-brain white matter. **b.** White matter coverage. **c.** Comparison of *R*_*rmse*_ across connectome models covering whole-brain. Error bar depicts ±1 s.e.m. across five individual brains. Conventions are identical to those in Fig 4. **d.** Maps of measured and predicted diffusion signal in a typical coronal brain slice for a single diffusion direction (subject 1, STN96 dataset). Colors indicate the normalized anisotropic diffusion signal for a single diffusion direction (red: higher signal, blue: lower signal). We plot the measured diffusion signal from two independent sessions as well as the diffusion signal prediction from two connectome models (SPC 0.25 mm and ETCpre).

Testing the ETC performance in the total white matter volume is computationally demanding, because of the increase of the matrix size in LiFE with ET (see the recent paper [49] for computational load of LiFE). For example, if we combine five whole-brain SPCs including 2 million streamlines, the candidate ETC size is 10 million streamlines. In order to generate whole-brain ETC model, we used the ETC-preselection method (see S1 Text, Section 5). Briefly, we selected streamlines from each SPC with highest weight (best contributing to predicting the diffusion signal) to build the candidate ETC. This ETC-preselection method reduces the size of the candidate ETC, but produces better prediction accuracy as compared with any SPC (S10 Fig). Using ETC-preselection method, we optimized the whole-brain ETCs in five brains (Fig 7).

We compared properties of preselected ETC with those of the SPCs. Consistent with results in occipital white matter (Figs 4 and 5), the whole-brain ETC supports a larger number of streamlines (Fig 7a), covers larger portion of white matter (Fig 7b) and predicts the diffusion signal better than any of the SPCs (Fig 7c). Fig 7d shows maps of measured (Data 1 and 2) and predicted diffusion signal for a single diffusion direction using two connectome models (SPC 0.25 mm and ETC with preselection). The result suggests that the ET approach is also effective for whole-brain connectome analysis.

### Robustness across datasets

We evaluated ET also using data from the Human Connectome Project (HCP90 [50]; see Materials and Methods). Consistent with results obtained on the STN96 data set, ET included a wider range of curvatures (S7b Fig), increased streamline count and white matter coverage (S7a and S7c Fig), and higher accuracy for predicting diffusion signal (S7d Fig). ETC on HCP90 dataset also supports example short- and long-range fascicles, U-fiber and the ILF (S7e Fig), as identified on the STN96 data. Thus, the properties of ET are consistent between these different datasets.

### Ensemble tractography across different algorithms and parameters

In addition to the ET method described above, we also used the ET method to create candidate connectomes that include streamlines from different algorithms (Tensor deterministic, CSD deterministic and CSD probabilistic in MRtrix [18]; see S1 Text, Section 3). The optimized connectomes from the ensemble of these algorithms had better prediction accuracy, and both increased streamline count and white matter coverage (S8 Fig). We also observed that the ETC generated using an ensemble of Fiber Orientation Distribution (FOD) amplitude cutoff parameters had better prediction accuracy as compared with SPCs (S9 Fig; S1 Text, Section 4). Hence, we find substantial evidence across different diffusion datasets, tractography methods and parameters sets that ET improves the connectome model.

## Discussion

We describe a new approach, Ensemble Tractography (ET), for estimating and selecting white matter fascicles from diffusion data in living human brains. This method addresses a widely recognized problem with tractography methods: tract estimates depend significantly on the choice of algorithm and parameters [32–40].

The ET method reduces the parameter and algorithm dependency by creating candidate connectomes whose tracts are generated using a range of parameters and algorithms. We illustrated ET for the case of sweeping out the curvature parameter in the MRtrix algorithm. We show that any single choice of the curvature parameter biases the distribution of candidate streamlines (Figs 3, S3, S4, and S7b), and that different parameter values are better suited for different types of fascicles (Figs 1 and 6). The candidate connectome is created as an ensemble, and the LiFE method is used to select an optimized connectome from the ensemble candidate connectome.

We have three principal findings. First, the optimized ensemble tractography connectome predicts diffusion signals better than any tested single parameter connectome. Second, the ensemble tractography connectome includes more unique streamlines and generates a denser representation than any single parameter connectomes. Third, the ensemble tractography connectome includes streamlines having different degree of curvature and length, and represent valuable anatomical features of the human white matter such as long- and short-range fibers.

### Alternative ET architectures

There is an enormous space of possible methods for creating candidate ETCs. The method for creating ensembles will need to evolve over many experiments from different laboratories. This paper presents one simple ET architecture that we found to be effective and efficient; just adding all streamlines from each parameter setting and optimize the ETC. One of the disadvantages of the ETC method presented in this paper is the computational demand required in building large candidate sets. In the following we discuss alternative architectures that we considered.

S1 Text (Section 5) proposes one alternative ET method; ETC-preselection. In this method, we chose 20% of streamlines contributing diffusion signal prediction from each of the individually optimized SPCs to build a new candidate ETC. The advantage of this method is that the resulting size of new candidate ETC becomes equal to that of original candidate SPCs. The disadvantage of this method is that we must evaluate (using LiFE) individually each SPC and also the ETC. Our results show that ETC-preselection performs significantly better than SPCs, and only slightly worse than ETC without preselections (S10 Fig). Preselection is particularly useful for whole-brain models including large streamline sets (Fig 7), but not necessarily the best for connectome models with smaller size.

As it is impossible to evaluate all possible ET algorithms in an initial paper, we describe the method and provide an open-source implementation (francopestilli.github.io/life/; github.com/brain-life/life/) to the community for exploration of the many possible options.

### Related literature

Several groups have analyzed tractography limitations, including parameter and algorithm dependence [32–40].

Bastiani and colleagues [34] analyzed how parameter and tractography algorithms influence connectomes and network properties. Their paper and others motivates the need for a means of deciding which solutions are best supported by the data [46,51–55] (see also [56]). Several other groups also noted that the best parameter differs between different white matter pathways [40,41].

BlueMatter [27] used streamlines generated by three different algorithms (STT [20], TEND [21], ConTrack [16]) to create a candidate connectome. An important difference is that the BlueMatter algorithm could only be run on a supercomputer (BlueGene/L, a 2048-processor supercomputer with 0.5 TB of memory), while the current ET algorithm using LiFE runs on a personal computer [49]. This advance enables investigators to systematically combine streamlines from many different parameters and algorithms and adopt ensemble tractography into their daily work flow. This paper is the first systematic exploration to sweep out several key parameters (curvature, stopping criterion) in tractography and demonstrate the advantage of ensemble methods in terms of anatomy (Fig 1) and prediction accuracy for diffusion signal (Figs 5 and 7).

A number of groups compared tractography with an independent measurement, such as invasive tract tracing or manganese enhanced MRI in macaques or mice [39,40,57–60]. For example, Thomas et al. [22] collected a diffusion data set in one macaque and compared the results of several single parameter connectomes with tracer measurements from a different macaque. This comparison has several limitations. First, the tracer measurements depend upon factors including the tracer type (e.g., anterograde or retrograde) and the selection of planes and injection sites; hence, they can differ substantially (e.g. [61,62]). When the methods disagree, it is often best to assemble a conclusion from multiple studies. Second, comparisons in a particular data set do not guarantee validation in a different experiment. For example, we cannot use high-resolution human adult brain fMRI data acquired in 7T scanner to support conclusions made from lower resolution fMRI data in children acquired using a 1.5 T scanner. Each methodology requires means for stating both the conclusions and the strength of the support for those conclusions. It is best to integrate fully justified findings derived by a variety of methods rather than discarding one method or another.

Others have proposed to evaluate tractography by defining ground truth using synthetic phantoms [31,63–66]. Some investigators have pointed out the logical limitations of this approach [5]. We agree that there are limitations to using phantoms for testing tractography but that in some cases synthetic phantoms can be valuable for analyzing computational methods. Unfortunately, for our current work none of the currently available phantoms can be used. This is because most phantoms have been generated using either single tractography parameters [67] or simple fiber configurations [63]. Close and colleagues [68] provide software for generating numerical phantoms that can simulate complex fiber organization. However, their method was not proposed to evaluate tractography performance by comparison with ground truth. This fact makes it impossible for us to use the current phantoms to test the superiority of multiple tractography approaches such as ET to resolving multiple types of fiber configurations simultaneously.

The potential value of creating connectomes from a collection of tractography methods was mentioned by both Sherbondy et al. [27] and Lemkaddem et al. [31]. Here, we provide a specific, open-source, implementation, and we begin a systematic analysis of this methodology. The analyses show that ET based on sweeping out the curvature parameter has the specific benefit of creating connectomes with both short- and long-range fascicles. In addition, the ET method produces more fascicles, larger coverage, and a better cross-validated prediction error.

### Future work

In this paper, we described the advantage of combining multiple tractography parameters and algorithms in order to improve the accuracy of connectome models. We use several example parameters and algorithms as a target for ET applications, and there are likely to be other beneficial combinations of algorithms and parameters which will be tested in future work. For example, we could combine connectomes by sweeping out two different parameters, or combine connectomes generated by different software packages that implement different algorithms, or combine connectome generated by using different seeding strategy tested in the literature [38,65,69]. Although it is impossible to test every pattern of combinations in this paper, we made LiFE software open (http://francopestilli.github.io/life/; https://github.com/brain-life/life/) to help other researchers test different ET architectures. Future studies by multiple research groups will clarify the optimal ET architecture in both model accuracy and computational efficiency.

ET will be generally applicable for many different proposed tractography algorithms. Several groups proposed generating streamlines based on the goodness-of-fit on the local diffusion signals (global tractography; [16,22–31,53]). While global tractography has advantages compared with local tractography [70], it too requires the user to set the parameters and this produces a parameter-selection dependency [34]. The ET approach will be effective in supporting both local and global tractography.

Current tractography uses a fixed set of parameters to generate each streamline. However, several fascicles, such as many within the optic radiation, include both curving and straight sections [71–74]. When this is known a priori, it may be more accurate to change the tractography parameter along one fascicles, allowing high and low curvature in the relevant portions of the tract. LiFE and ET will provide the opportunity to evaluate the model accuracy of new tractography tools in terms of the prediction accuracy on diffusion signal.

### Extending the range of tractography

It is widely agreed that diffusion MRI contributes useful information about the large and long-range fasciculi in the human brain [75–78]. Meanwhile, the existence of U-fiber system has been supported [79,80], but not extensively studied in the literature presumably because of the limitations in tractography parameter selections. The optimized ETCs extend tractography to include both long- and short-range fascicles in a single connectome, improving on the optimized SPCs which include one or the other. The higher model-accuracy and the inclusion of both short- and long-range fibers is a validation that the optimized ETC improves on any SPC. The preliminary ET results are encouraging, but they will surely benefit from further optimization.

Tracer studies are not well-suited to identifying long-range pathways in the human brain. Even in animal models, with more than a century of history, recent tracer measurements challenge conventional thinking about long-range pathways. Reports describing many new found projections demonstrate that the field is active and evolving [62,81,82].

The progress in human tractography complements the strengths of tracer studies in animal models. Ultimately, combining insights from these technologies will provide a more complete view of human brain anatomy and function.

## Materials and Methods

### MR data acquisition and pre-processing

We used two magnetic resonance diffusion imaging datasets. The STN96 dataset was acquired at the Stanford Center for Neurobiological Imaging (CNI); the HCP90 dataset was acquired by the Human Connectome Consortium [50].

#### STN96 data set: Diffusion-weighted MRI acquisition

The main analyses were conducted for the STN96 dataset. These have also been used in other papers [46–49]. STN96 was collected from five human subjects (five males; age range 27–40, mean age 32.6 years old). Informed written consent was obtained from all subjects. The experimental procedures were approved by the Stanford University Institutional Review Board.

A dual spin echo diffusion-weighted sequence [83] was used. The diffusion MRI data were acquired for 96 different directions at a spatial resolution of 1.5 mm^3^ (isotropic), two averages in k-space (i.e., NEX = 2). The b-value was 2000 s/mm^2^ and TE was 96.8 msec. Ten non-diffusion weighted images (b = 0) were acquired at the beginning of each scan. Two scans were performed.

MR images were corrected for subject’s motion using a rigid body alignment algorithm [84]. We also used the measurements of the B_0_ magnetic field for post-hoc correction of EPI spatial distortion (https://github.com/kendrickkay/preprocessfmri). Dual-spin echo sequence minimizes the eddy-current artifact [83]. Hence, eddy current correction was not applied. All pre-processing steps have been implemented in Matlab as part of the mrVista software distribution (https://github.com/vistalab/vistasoft).

#### HCP90 data set

The HCP90 data set was acquired at multiple b-values (1000, 2000 and 3000 s/mm^2^). Measurements from the 2000 s/mm^2^ shell were extracted from the original data set and used for analyses because the implementation of LiFE that we used only accepts single-shell diffusion MRI data [46]. Processing methods for HCP data has been described elsewhere [85,86].

### Selection and evaluation of white-matter connectomes

#### Candidate connectome generation

The total white-matter volume was initially identified from the tissue type segmentation using FreeSurfer [87], edited manually ([88] http://www.itksnap.org/pmwiki/pmwiki.php), and finally resampled at the resolution of the diffusion data. Portions of the white-matter volume were used as seed regions for fiber tracking. S2 Fig depicts occipital white matter regions (10 hemispheres) used for the main analyses in STN96 dataset. Whereas most of the analyses on the STN96 dataset were focused on the occipital white matter, we also used the total white matter volume for testing the generality of the findings (see Fig 7).

The candidate connectome was created using fiber tracking in MRtrix 0.2 [18]. We used a constrained-spherical deconvolution (CSD [89]) and probabilistic tracking (step size: 0.2 mm; maximum length: 200 mm; minimum length: 10 mm; FOD amplitude stopping criterion: 0.1; vector specifying the initial direction: 20 deg). We set the maximum number of spherical harmonics to 8 (*L*_*max*_ = 8). We used the entire total white matter mask as seed, and seed voxels were randomly chosen from the mask for producing individual streamlines. Tracking was terminated when a streamline reached outside the white matter mask. The minimum radius of curvature was set to different values in different candidate connectomes, comprising the ensemble.

In both datasets, we initially performed whole-brain tracking to generate 2 million streamlines for each parameter settings. For the analysis using occipital white matter, we clipped the streamlines at the boundary of white matter Region of Interest (ROI) described in S2 Fig.

For the STN96 dataset, each subject had two scans; one was used to create the candidate connectomes and the second was used for cross-validation (see “Evaluation of model accuracy” below).

#### Connectome model optimization and evaluation

We optimized connectome models using LiFE (Linear Fascicle Evaluation [46], https://francopestilli.github.io/life/; https://github.com/brain-life/life/). Briefly, LiFE uses the candidate connectome to create a linear model that predicts the measured diffusion signal. From the linear model, LiFE derives a weight describing each streamline’s contribution to predicting the data. The weight is estimated using a non-negative least-square optimization method (SBB [90]). The model accuracy is assessed by using the model to predict a diffusion data set. The evaluation is global in that the error is measured for the entire set of streamlines and the entire diffusion MRI data set. The processing of one occipital connectome model (160,000 streamlines) requires 64.7 minutes on the computer we used to analyze STN96 dataset (16 processing core with 32GB Random Access Memory). The computational load of LiFE on standard notebook computer is described elsewhere [49].

We evaluated two types of connectomes:

Single parameter connectome (SPC): Connectome model generated by a single curvature parameter. We generated four connectome models by using five different curvature parameters (the minimum radius of curvature = 0.25, 0.5, 1, 2 and 4 mm). These curvature parameters correspond to angle thresholds 47.2 deg, 23.1 deg, 11.5 deg, 5.7 deg and 2.9 deg respectively, in a step size (0.2 mm) we used (see S11 Fig for the relation between minimum radius of curvature and angle). In each SPC models, we used 160,000 streamlines as candidate connectome for occipital white matter regions used for each analysis.
Ensemble tractography connectome (ETC): Connectome model generated from multiple curvature parameters. The candidate connectome streamlines derive from five SPC models, and each SPC include 160,000 streamlines as described above. Thus, the candidate ETC connectome includes 800,000 streamlines. Fig 2 describes the flowchart of the ETC. Alternatives to the ETC that include preselection are described in S1 Text, Section 5.

#### Evaluation of model accuracy

Model accuracy is evaluated by comparing the error between the LiFE model prediction and the test-retest reliability. Specifically, we evaluated the model prediction error using cross-validation in order to control over-fitting [46,91]. We compute this error in a series of simple calculations [46].

First, we calculate the model root mean squared error (RMSE), *M*_*rmse*_, as:

$$M_{rmse}=\;\sqrt{\frac{\sum_{i=1}^{N}{\left(m\left(\theta_{i}\right)-S_{2}\left(\theta_{i}\right)\right)}^{2}}{N}}$$ (1)

Where *m*(*θ*_*i*_) is the diffusion-modulation predicted by connectome model at each measured diffusion direction*θ*_*i*_ and *S*_2_(*θ*_*i*_) is the measured diffusion-modulation signal in a second, independent set of diffusion data not used for tractography. *N* is a number of measured diffusion directions.

Second, we calculate the test-retest reliability, *D*_*rmse*_, from the repeated measurements.

$$D_{rmse}=\;\sqrt{\frac{\sum_{i=1}^{N}{\left(S_{1}\left(\theta_{i}\right)-S_{2}\left(\theta_{i}\right)\right)}^{2}}{N}}$$ (2)

The signals *S*_1_(*θ*_*i*_) and *S*_2_(*θ*_*i*_) are two diffusion-weighted measurements in the same subject.

Finally, model accuracy is analyzed as the ratio of the prediction to the test-retest reliability, *R*_*rmse*_:

$$R_{rmse}=\frac{M_{rmse}}{D_{rmse}}$$ (3)

A value of *R*_*rmse*_ = 1 indicates that the optimized connectome model predicts the second data as accurately as test-retest reliability. We evaluated the accuracy of each connectome model by using the *R*_*rmse*_ (Eq 3) to describe how well model predicts an independent dataset (cross-validation) with respect to the noise in the STN96 dataset (test-retest reliability). The theoretical lower bound of *R*_*rmse*_ is 0.707 [48].

The HCP data set does not include a second independent scan. Hence, for this data set, we used the RMSE between diffusion signal prediction and first diffusion data for evaluating connectome model accuracy (S7d Fig). This number has no absolute significance, but it can be used to compare relative model performance for model fits to data sets. More technical details about LiFE have been published [46,47,49].

#### Measuring mean streamline curvature

We computed the streamline curvature distribution in each connectome model. First, we fit a spline function to individual streamlines. We then computed extrinsic curvature (*C*) using individual spline curves at individual step points:

$$C=\frac{\sqrt{\left({\left(x^{\prime \prime }y^{\prime }-x^{\prime }y^{\prime \prime }\right)}^{2}+{\left(x^{\prime \prime }z^{\prime }-x^{\prime }z^{\prime \prime }\right)}^{2}+{\left(x^{\prime \prime }z^{\prime }-x^{\prime }z^{\prime \prime }\right)}^{2}\right)}}{{\left({x^{\prime }}^{2}+{y^{\prime }}^{2}+{z^{\prime }}^{2}\right)}^{\frac{3}{2}}}$$ (4)

Where *x'*, *y'*, *z'* and *x"*, *y"*, *z"* are the first and second derivative respectively of the *x*, *y*, *z* oordinates at each node in a streamline. We computed the mean curvature $\overset{-}{C}$ across all nodes in the streamline as follows:

$$\bar{C}\;=\frac{\;\sum_{i=1}^{N}C_{i}}{N}$$ (5)

where *N* is the number of nodes along the streamlines. The mean radius of curvature was defined as the inverse of mean curvature:

$${\text{r}}_{\bar{C}}\;={\bar{C}}^{\;-1}$$ (6)

We computed the mean radius of curvature in all streamlines and plotted the distribution in Figs 3 (STN96 data set) and S7b (HCP90 data set). This is the same computation used in MRtrix to generate the streamlines given a certain parameter [18].

### Tract identification

We identified several tracts within each optimized connectome to compare how different connectome represents anatomical features of the white-matter fascicles. All figures of brain anatomy and fascicles were made using Matlab Brain Anatomy (www.github.com/francopestilli/mba).

#### Inferior Longitudinal Fasciculus

We identified ILF in one subject in STN96 dataset (subject 1, left hemisphere) and one subject in HCP90 dataset (subject 6, left hemisphere). We used the AFQ toolbox [38] to identify ILF from connectome models. Briefly, AFQ defined waypoint ROIs in individual subject by non-linear transformation from waypoint ROI in MNI template brain, which is drawn on the basis of anatomical prescription [75]. ILF is identified as streamlines passing through two waypoint ROIs. We excluded streamlines with length ≥ 3 sd and with position ≥ 3 sd away from the mean position of the ILF [76].

#### U-fiber in occipital cortex

We identified U-fiber system (a fascicle set travelling parallel to a cortical sulcus; [79]) in occipital cortex in one subject in STN96 dataset (subject 1, left hemisphere) and one subject in HCP90 dataset (subject 6, left hemisphere). We manually defined two waypoint ROIs to identify U-fibers from connectome models (the location of ROIs is shown in S1 Fig). We selected the streamlines having endpoints in both of these ROIs in all connectome models as U-fibers. We excluded topological outliers based on the length and position, by using the same criterion for ILF. Example result is shown in Fig 1. In subject 1 in STN96 dataset, the comparison with visual field maps [92,93] showed that this U-fiber is connecting V3A/B and V3d.

### Fascicle evaluation for whole-brain connectome

We evaluated model accuracy for whole-brain connectomes. To do so, we generated five 2-million streamlines candidate SPCs by using different curvature thresholds (from 0.25 mm to 4 mm). We then used LiFE to assign a weight to each streamline. Next, we selected the top 400,000 streamlines with highest weight from each SPC (preselection method; see S1 Text, Section 5). This resulted in an ETC connectome containing 2 million streamlines. Finally, we optimized this ETC using LiFE. The processing of one whole-brain connectome model with 2 million streamlines requires 28.4 hours on a computer with 16 processing cores and 32GB Random Access Memory.

### Fascicle evaluation along the ILF

The ILF extends outside the occipital white matter region used for the main analysis (S2 Fig). In order to evaluate the connectome model along these fascicles, we selectively fitted LiFE to white matter voxels containing these tracts. To do so, we (1) identified the ILF from candidate connectome in all connectome models using the identification method described above, (2) concatenated all streamlines identified as ILF across multiple connectome models, (3) extracted the voxels in which any of streamlines are passing through. Finally we obtained a white matter region covering the ILF. LiFE analysis on the ILF is limited to these portions of white matter in all connectome models tested.

## Supporting Information

S1 Text Supplementary methods. (DOCX)

S1 Fig Waypoint ROIs for identifying the U-fiber in occipital white matter. Colored regions depict two white matter regions used for identifying U-fiber. Streamlines having one endpoint for each ROI are identified as U-fiber. **a.** Subject 1, left hemisphere in STN96 dataset. **b.** Subject 6, left hemisphere in HCP90 dataset.
(EPS)

S2 Fig Occipital white matter regions used for the analysis for STN96 dataset. White matter regions used for the main analysis (Figs 3–5) in left (red) and right (blue) hemisphere from 5 subjects in STN96 dataset. The boundary of the white matter regions in each hemisphere is described in ACPC coordinate.
(EPS)

S3 Fig Streamline curvature distribution of candidate connectomes. Distribution of the radius of curvature in candidate connectome in six connectome models. Conventions are identical to Fig 3.
(EPS)

S4 Fig Streamline curvature distribution of whole-brain connectomes generated by PICo. Distribution of the radius of curvature in candidate connectome in four different whole-brain connectome, each of which is generated by using four different angle threshold (5.7, 11,5, 23.1, 47.2 deg) in PICo algorithm [11] on Camino toolbox (see S1 Text, Section 1). We have also observed that the connectome using lenient bound on the curvature (e.g. 47.2 deg angle threshold) does not produce straight streamlines having large radius of curvature. Plot conventions are identical to S3 Fig.
(EPS)

S5 Fig Example of crossing fascicle voxel in which ETC shows optimal performance. **a.** Measured and predicted diffusion signal from one example voxel (from Subject 5, STN96 dataset). Horizontal axis depicts the diffusion gradient directions (arbitrary order) and vertical axis depicts the magnitude of demeaned diffusion signal in each direction. Black lines depict measured diffusion signal (solid line, scan 1; dotted line, scan 2) whereas colored lines depict predicted diffusion signal (top panel, ETC; bottom panel; SPC 2 mm). Whereas the ETC predicts the diffusion signals, the SPC 2 mm fails. *R*_*rmse*_ in each plot indicates the *R*_*rmse*_ of ETC and SPC 2 mm model in the voxel. **b.** Spatial distribution of measured and predicted diffusion signal. Horizontal and vertical axis depicts the magnitude of demeaned diffusion signal in X and Z direction. Individual data points describe the measured or predicted demeaned diffusion signal in one of 96 diffusion-weighted directions. The plot indicates that ETC successfully predicts complex diffusion signal distribution derived from crossing fascicles. **c.** Scatter plot showing the correlation between measured and predicted diffusion signal. Horizontal axis depicts the prediction for demeaned diffusion signal by ETC (top panel) and SPC 2 mm (bottom panel). Vertical axis depicts the measured diffusion signal in diffusion dataset not used for tractography (cross-validation, see Materials and Methods). While ETC diffusion predictions showed a substantial correlation with the signal in independent dataset (*r* = 0.837), diffusion signal prediction by SPC 2mm does not correlate with diffusion signal (*r* = -0.015).
(EPS)

S6 Fig Comparison between ETC and SPC using large candidate connectome size. **a**. Flow diagram of BigSPC model. We generate the identical number of streamlines to ETC only using single parameter (minimum radius of curvature = 2 mm), and optimized it using LiFE (see S1 Text, Section 2). **b.** Optimized connectome size. BigSPC supports comparable number of streamlines to ETC. **c.** White matter coverage. ETC covers larger portion of white matter that the BigSPC. **d.** Comparison of model accuracy (*R*_*rmse*_) between ETC and BigSPC model. ETC predicts the diffusion signal better than the BigSPC. **e.** Comparison of *R*_*rmse*_ in an individual hemisphere. Each dot showed *R*_*rmse*_ of BigSPC and ETC in individual hemispheres. The results indicate that ETC showed the better performance in most hemispheres.
(EPS)

S7 Fig Comparison of connectome models from Human Connectome Project (HCP) data. **a.** Optimal connectome size in 8 hemispheres from HCP90 dataset (occipital cortex). Error bar depicts ±1 s.e.m. across hemispheres. Conventions are identical to those in Fig 4a and 4**b.** Distribution of radius of curvature in optimized connectome in six connectome models (SPCs and ETC), averaged across 8 hemispheres. Conventions are identical to those in Fig 3. **c.** White matter coverage. **d.** Comparison of Root Mean Squared Error (RMSE) between measured and predicted diffusion signal across connectome models. Other conventions are identical to those in Fig 5b. **e.** U-fiber and ILF supported by different connectome models. In SPC model using 2mm, there are no streamlines in the optimized connectome projecting two gyri in dorsal visual cortex. Conventions are identical to Fig 1.
(TIF)

S8 Fig Ensemble Tractography using multiple tractography algorithms. **a.** Flow diagram of Ensemble Tractography across algorithms (see S1 Text, Section 3). Using three different tractography algorithms in MRtrix (DT_STREAM: Tensor deterministic; SD_STREAM: CSD deterministic, and SD_PROB: CSD probabilistic; [18]), we generated three Single Algorithm Connectome (SAC) candidate containing 120,000 streamlines in occipital cortex. In ETC model, we simply combined all SAC streamlines into ETC candidate connectome. We used LiFE to optimize SACs and ETC. **b.** Optimized connectome size of four connectome models. **c.** White matter coverage. **d.** Accuracy of ETC. ETC predicts diffusion signal better than SACs. Conventions are identical to those in S6 Fig.
(EPS)

S9 Fig Ensemble Tractography comparing across stopping criterion. **a.** Flow diagram of Ensemble Tractography across Fiber Orientation Distribution (FOD) amplitude stopping criterion (see S1 Text, Section 4). Using four different FOD amplitude stopping criterions (0, 0.05, 0.1 and 0.2) in MRtrix, we generated four SPCs containing 160,000 streamlines in occipital cortex. In ETC model, we simply combined four SPCs to generate candidate connectome. We used LiFE to optimize each connectome model. **b.** Optimized connectome size of four connectome models. **c.** White matter coverage. **d.** Accuracy of ETC. ETC predicts diffusion signal better than SPCs. Conventions are identical to those in S6 Fig.
(EPS)

S10 Fig ETC-preselection model. **a.** Flow diagram of the ETC-preselection (‘ETCpre’; see S1 Text, Section 5). Using LiFE, we optimize each SPC first, and select streamlines contributing diffusion signal prediction in each SPC. We combine those preselected streamlines to create candidate ETCpre connectome, and optimized it using LiFE again. See S1 Text, Section 5 for detail. **b.** Optimized connectome size of SPCs, ETCpre and ETC. The optimized ETCpre supports larger number of streamlines as compared to SPCs, meanwhile candidate connectome size is identical. **c.** White matter coverage. ETCpre covers wider regions of white matter as compared with SPCs. **d.** Accuracy of ETCpre. ETCpre predicts diffusion signal better than SPCs. Accuracy is slightly lower than ETC without preselection. Conventions are identical to those in S6 Fig.
(EPS)

S11 Fig The relation between curvature and angle threshold in tractography. The plot describes the relationship between the minimum radius of curvature and angle threshold when step size is 0.2 mm, based on the formula in Tournier et al. (2012) [18].
(EPS)

## References

[1] Mori S, Zhang J (2006) Principles of diffusion tensor imaging and its applications to basic neuroscience research. Neuron 51: 527–539. 16950152 10.1016/j.neuron.2006.08.012

[2] Craddock RC, Jbabdi S, Yan CG, Vogelstein JT, Castellanos FX, Di Martino A, et al (2013) Imaging human connectomes at the macroscale. Nature Methods 10: 524–539. 10.1038/nmeth.2482 23722212 PMC4096321

[3] Thomason ME, Thompson PM (2011) Diffusion imaging, white matter, and psychopathology. Annu Rev Clin Psychol 7: 63–85. 10.1146/annurev-clinpsy-032210-104507 21219189

[4] Wandell BA, Yeatman JD (2013) Biological development of reading circuits. Curr Opin Neurobiol 23: 261–268. 10.1016/j.conb.2012.12.005 23312307 PMC3622751

[5] Jbabdi S, Johansen-Berg H (2011) Tractography: where do we go from here? Brain Connect 1: 169–183. 10.1089/brain.2011.0033 22433046 PMC3677805

[6] Jones DK, Knosche TR, Turner R (2013) White matter integrity, fiber count, and other fallacies: the do's and don'ts of diffusion MRI. Neuroimage 73: 239–254. 10.1016/j.neuroimage.2012.06.081 22846632

[7] Felleman DJ, Van Essen DC (1991) Distributed hierarchical processing in the primate cerebral cortex. Cereb Cortex 1: 1–47. 1822724 10.1093/cercor/1.1.1-a

[8] Sporns O, Tononi G, Kotter R (2005) The human connectome: A structural description of the human brain. PLoS Comput Biol 1: e42 16201007 10.1371/journal.pcbi.0010042 PMC1239902

[9] Hagmann P, Cammoun L, Gigandet X, Gerhard S, Grant PE, Wedeen V, et al (2010) MR connectomics: Principles and challenges. J Neurosci Methods 194: 34–45. 10.1016/j.jneumeth.2010.01.014 20096730

[10] Mori S, Crain BJ, Chacko VP, van Zijl PC (1999) Three-dimensional tracking of axonal projections in the brain by magnetic resonance imaging. Ann Neurol 45: 265–269. 9989633 10.1002/1531-8249(199902)45:2<265::aid-ana21>3.0.co;2-3

[11] Parker GJM, Haroon HA, Wheeler-Kingshott CAM (2003) A framework for a streamline-based probabilistic index of connectivity (PICo) using a structural interpretation of MRI diffusion measurements. Journal of Magnetic Resonance Imaging 18: 242–254. 12884338 10.1002/jmri.10350

[12] Behrens TE, Woolrich MW, Jenkinson M, Johansen-Berg H, Nunes RG, Clare S, et al (2003) Characterization and propagation of uncertainty in diffusion-weighted MR imaging. Magn Reson Med 50: 1077–1088. 14587019 10.1002/mrm.10609

[13] Jiang H, van Zijl PCM, Kim J, Pearlson GD, Mori S (2006) DtiStudio: Resource program for diffusion tensor computation and fiber bundle tracking. Computer Methods and Programs in Biomedicine 81: 106–116. 16413083 10.1016/j.cmpb.2005.08.004

[14] Cook PA, Bai Y, Nedjati-Gilani S, Seunarine KK, Hall MG, Parker GJ, et al (2006) Camino: Open-Source Diffusion-MRI Reconstruction and Processing. Proc Intl Soc Mag Reson Med: 2759.

[15] Wang R, Benner T, Sorensen AG, Wedeen VJ (2007) Diffusion Toolkit: A Software Package for Diffusion Imaging Data Processing and Tractography. Proc Intl Soc Mag Reson Med 15: 3720.

[16] Sherbondy AJ, Dougherty RF, Ben-Shachar M, Napel S, Wandell BA (2008) ConTrack: finding the most likely pathways between brain regions using diffusion tractography. J Vis 8: 15 11–16. 10.1167/8.9.15 PMC2696074 18831651

[17] Leemans A, Jeurissen B, Sijbers J, Jones DK (2009) ExploreDTI: a graphical toolbox for processing, analyzing, and visualizing diffusion MR data. Proc Intl Soc Mag Reson Med: 3537.

[18] Tournier JD, Calamante F, Connelly A (2012) MRtrix: Diffusion tractography in crossing fiber regions. International Journal of Imaging Systems and Technology 22: 53–66.

[19] Conturo TE, Lori NF, Cull TS, Akbudak E, Snyder AZ, Shimony JS, et al (1999) Tracking neuronal fiber pathways in the living human brain. Proc Natl Acad Sci U S A 96: 10422–10427. 10468624 10.1073/pnas.96.18.10422 PMC17904

[20] Basser PJ, Pajevic S, Pierpaoli C, Duda J, Aldroubi A (2000) In vivo fiber tractography using DT-MRI data. Magn Reson Med 44: 625–632. 11025519 10.1002/1522-2594(200010)44:4<625::aid-mrm17>3.0.co;2-o

[21] Lazar M, Weinstein DM, Tsuruda JS, Hasan KM, Arfanakis K, Meyerand ME, et al (2003) White matter tractography using diffusion tensor deflection. Hum Brain Mapp 18: 306–321. 12632468 10.1002/hbm.10102 PMC6871932

[22] Poupon C, Clark CA, Frouin V, Regis J, Bloch I, Le Bihan D, et al (2000) Regularization of diffusion-based direction maps for the tracking of brain white matter fascicles. Neuroimage 12: 184–195. 10913324 10.1006/nimg.2000.0607

[23] Mangin JF, Poupon C, Cointepas Y, Riviere D, Papadopoulos-Orfanos D, Clark CA, et al (2002) A framework based on spin glass models for the inference of anatomical connectivity from diffusion-weighted MR data—a technical review. NMR Biomed 15: 481–492. 12489097 10.1002/nbm.780

[24] Iturria-Medina Y, Canales-Rodriguez EJ, Melie-Garcia L, Valdes-Hernandez PA, Martinez-Montes E, Aleman-Gomez Y, et al (2007) Characterizing brain anatomical connections using diffusion weighted MRI and graph theory. Neuroimage 36: 645–660. 17466539 10.1016/j.neuroimage.2007.02.012

[25] Kreher BW, Mader I, Kiselev VG (2008) Gibbs tracking: a novel approach for the reconstruction of neuronal pathways. Magn Reson Med 60: 953–963. 10.1002/mrm.21749 18816816

[26] Jbabdi S, Bellec P, Toro R, Daunizeau J, Pelegrini-Issac M, Benali H (2008) Accurate anisotropic fast marching for diffusion-based geodesic tractography. Int J Biomed Imaging 2008: 320195 10.1155/2008/320195 18299703 PMC2235929

[27] Sherbondy AJ, Dougherty RF, Ananthanarayanan R, Modha DS, Wandell BA (2009) Think global, act local; projectome estimation with BlueMatter. Med Image Comput Comput Assist Interv 12: 861–868. 20426069 10.1007/978-3-642-04268-3_106 PMC3076280

[28] Fillard P, Poupon C, Mangin JF (2009) A novel global tractography algorithm based on an adaptive spin glass model. Med Image Comput Comput Assist Interv 12: 927–934. 10.1007/978-3-642-04268-3_114 20426077

[29] Sotiropoulos SN, Bai L, Morgan PS, Constantinescu CS, Tench CR (2010) Brain tractography using Q-ball imaging and graph theory: Improved connectivities through fibre crossings via a model-based approach. Neuroimage 49: 2444–2456. 10.1016/j.neuroimage.2009.10.001 19818861

[30] Reisert M, Mader I, Anastasopoulos C, Weigel M, Schnell S, Kiselev V (2011) Global fiber reconstruction becomes practical. Neuroimage 54: 955–962. 10.1016/j.neuroimage.2010.09.016 20854913

[31] Lemkaddem A, Skioldebrand D, Dal Palu A, Thiran JP, Daducci A (2014) Global tractography with embedded anatomical priors for quantitative connectivity analysis. Front Neurol 5: 232 10.3389/fneur.2014.00232 25452742 PMC4233943

[32] Parizel PM, Van Rompaey V, Van Loock R, Van Hecke W, Van Goethem JW, Leemans A, et al (2007) Influence of user-defined parameters on diffusion tensor tractography of the corticospinal tract. Neuroradiol J 20: 139–147. 24299634 10.1177/197140090702000202

[33] Taoka T, Morikawa M, Akashi T, Miyasaka T, Nakagawa H, Kiuchi K, et al (2009) Fractional anisotropy-threshold dependence in tract-based diffusion tensor analysis: evaluation of the uncinate fasciculus in Alzheimer disease. AJNR Am J Neuroradiol 30: 1700–1703. 10.3174/ajnr.A1698 19541775 PMC7051508

[34] Bastiani M, Shah NJ, Goebel R, Roebroeck A (2012) Human cortical connectome reconstruction from diffusion weighted MRI: the effect of tractography algorithm. Neuroimage 62: 1732–1749. 10.1016/j.neuroimage.2012.06.002 22699045

[35] Domin M, Langner S, Hosten N, Lotze M (2014) Comparison of parameter threshold combinations for diffusion tensor tractography in chronic stroke patients and healthy subjects. PLoS One 9: e98211 10.1371/journal.pone.0098211 24853163 PMC4031143

[36] Kunimatsu A, Aoki S, Masutani Y, Abe O, Hayashi N, Mori H, et al (2004) The optimal trackability threshold of fractional anisotropy for diffusion tensor tractography of the corticospinal tract. Magn Reson Med Sci 3: 11–17. 16093615 10.2463/mrms.3.11

[37] Stadlbauer A, Nimsky C, Buslei R, Salomonowitz E, Hammen T, Buchfelder M, et al (2007) Diffusion tensor imaging and optimized fiber tracking in glioma patients: Histopathologic evaluation of tumor-invaded white matter structures. Neuroimage 34: 949–956. 17166744 10.1016/j.neuroimage.2006.08.051

[38] Li L, Rilling JK, Preuss TM, Glasser MF, Hu X (2012) The effects of connection reconstruction method on the interregional connectivity of brain networks via diffusion tractography. Hum Brain Mapp 33: 1894–1913. 10.1002/hbm.21332 21928316 PMC6414228

[39] Azadbakht H, Parkes LM, Haroon HA, Augath M, Logothetis NK, de Crespigny A, et al (2015) Validation of High-Resolution Tractography Against In Vivo Tracing in the Macaque Visual Cortex. Cereb Cortex: Epub ahead of print. 10.1093/cercor/bhu326 PMC4816782 25787833

[40] Thomas C, Ye FQ, Irfanoglu MO, Modi P, Saleem KS, Leopold DA, et al (2014) Anatomical accuracy of brain connections derived from diffusion MRI tractography is inherently limited. Proc Natl Acad Sci U S A 111: 16574–16579. 10.1073/pnas.1405672111 25368179 PMC4246325

[41] Chamberland M, Whittingstall K, Fortin D, Mathieu D, Descoteaux M (2014) Real-time multi-peak tractography for instantaneous connectivity display. Front Neuroinform 8: 59 10.3389/fninf.2014.00059 24910610 PMC4038925

[42] Dasarathy BV, Sheela BV (1979) Composite classifier system design: concepts and methodology. Proceedings of the IEEE 67: 708–713.

[43] Dietterich TG (2000) Ensemble methods in machine learning. Multiple Classifier Systems 1857: 1–15.

[44] Drucker H, Cortes C, Jackel LD, Lecun Y, Vapnik V (1994) Boosting and Other Ensemble Methods. Neural Computation 6: 1289–1301.

[45] Polikar R (2006) Ensemble based systems in decision making. Circuits and Systems Magazine, IEEE 6: 21–45.

[46] Pestilli F, Yeatman JD, Rokem A, Kay KN, Wandell BA (2014) Evaluation and statistical inference for human connectomes. Nat Methods 11: 1058–1063. 10.1038/nmeth.3098 25194848 PMC4180802

[47] Takemura H, Rokem A, Winawer J, Yeatman JD, Wandell BA, Pestilli F (2015) A major human white-matter pathway between dorsal and ventral visual cortex. Cereb Cortex: Epub ahead of print. 10.1093/cercor/bhv064 PMC4830295 25828567

[48] Rokem A, Yeatman JD, Pestilli F, Kay KN, Mezer A, van der Walt S, et al (2015) Evaluating the accuracy of diffusion MRI models in white matter. PLoS One 10: e0123272 10.1371/journal.pone.0123272 25879933 PMC4400066

[49] Caiafa CF, Pestilli F (2015) Sparse multiway decomposition for analysis and modeling of diffusion imaging and tractography. ArXiv: 1505.0710.

[50] Van Essen DC, Smith SM, Barch DM, Behrens TE, Yacoub E, Ugurbil K, et al (2013) The WU-Minn Human Connectome Project: an overview. Neuroimage 80: 62–79. 10.1016/j.neuroimage.2013.05.041 23684880 PMC3724347

[51] Smith RE, Tournier JD, Calamante F, Connelly A (2013) SIFT: Spherical-deconvolution informed filtering of tractograms. Neuroimage 67: 298–312. 10.1016/j.neuroimage.2012.11.049 23238430

[52] Smith RE, Tournier JD, Calamante F, Connelly A (2015) The effects of SIFT on the reproducibility and biological accuracy of the structural connectome. Neuroimage 104: 253–265. 10.1016/j.neuroimage.2014.10.004 25312774

[53] Daducci A, Dal Palu A, Alia L, Thiran JP (2014) COMMIT: Convex Optimization Modeling for Micro-structure Informed Tractography. IEEE Trans Med Imaging 34: 246–257. 10.1109/TMI.2014.2352414 25167548

[54] Smith RE, Tournier JD, Calamante F, Connelly A (2015) SIFT2: Enabling dense quantitative assessment of brain white matter connectivity using streamlines tractography. Neuroimage 119: 338–351. 10.1016/j.neuroimage.2015.06.092 26163802

[55] Schreiber J, Riffert T, Anwander A, Knosche TR (2014) Plausibility Tracking: a method to evaluate anatomical connectivity and microstructural properties along fiber pathways. Neuroimage 90: 163–178. 10.1016/j.neuroimage.2014.01.002 24418503

[56] Bassett DS, Brown JA, Deshpande V, Carlson JM, Grafton ST (2011) Conserved and variable architecture of human white matter connectivity. Neuroimage 54: 1262–1279. 10.1016/j.neuroimage.2010.09.006 20850551

[57] Dyrby TB, Sogaard LV, Parker GJ, Alexander DC, Lind NM, Baare WFC, et al (2007) Validation of in vitro probabilistic tractography. Neuroimage 37: 1267–1277. 17706434 10.1016/j.neuroimage.2007.06.022

[58] Dauguet J, Peled S, Berezovskii V, Delzescaux T, Warfield SK, Born R, et al (2007) Comparison of fiber tracts derived from in-vivo DTI tractography with 3D histological neural tract tracer reconstruction on a macaque brain. Neuroimage 37: 530–538. 17604650 10.1016/j.neuroimage.2007.04.067

[59] Jbabdi S, Lehman JF, Haber SN, Behrens TE (2013) Human and Monkey Ventral Prefrontal Fibers Use the Same Organizational Principles to Reach Their Targets: Tracing versus Tractography. Journal of Neuroscience 33: 3190–3201. 10.1523/JNEUROSCI.2457-12.2013 23407972 PMC3602794

[60] Calabrese E, Badea A, Cofer G, Qi Y, Johnson GA (2015) A Diffusion MRI Tractography Connectome of the Mouse Brain and Comparison with Neuronal Tracer Data. Cereb Cortex: Epub ahead of print. 10.1093/cercor/bhv121 PMC4715247 26048951

[61] Lyon DC, Connolly JD (2012) The case for primate V3. Proc Biol Sci 279: 625–633. 10.1098/rspb.2011.2048 22171081 PMC3248742

[62] Kennedy H, Knoblauch K, Toroczkai Z (2013) Why data coherence and quality is critical for understanding interareal cortical networks. Neuroimage 80: 37–45. 10.1016/j.neuroimage.2013.04.031 23603347

[63] Fillard P, Descoteaux M, Goh A, Gouttard S, Jeurissen B, Malcolm J, et al (2011) Quantitative evaluation of 10 tractography algorithms on a realistic diffusion MR phantom. Neuroimage 56: 220–234. 10.1016/j.neuroimage.2011.01.032 21256221

[64] Cote MA, Girard G, Bore A, Garyfallidis E, Houde JC, Descoteaux M (2013) Tractometer: towards validation of tractography pipelines. Med Image Anal 17: 844–857. 10.1016/j.media.2013.03.009 23706753

[65] Girard G, Whittingstall K, Deriche R, Descoteaux M (2014) Towards quantitative connectivity analysis: reducing tractography biases. Neuroimage 98: 266–278. 10.1016/j.neuroimage.2014.04.074 24816531

[66] Neher PF, Laun FB, Stieltjes B, Maier-Hein KH (2014) Fiberfox: facilitating the creation of realistic white matter software phantoms. Magn Reson Med 72: 1460–1470. 10.1002/mrm.25045 24323973

[67] Neher P, Houde JC, Caruyer E, Daducci A, Dyrby T, Maier-Hein K, et al (2015) ISMRM 2015 Tractography challenge. http://www.tractometer.org/ismrm_2015_challenge/

[68] Close TG, Tournier JD, Calamante F, Johnston LA, Mareels I, Connelly A (2009) A software tool to generate simulated white matter structures for the assessment of fibre-tracking algorithms. Neuroimage 47: 1288–1300. 10.1016/j.neuroimage.2009.03.077 19361565

[69] Smith RE, Tournier JD, Calamante F, Connelly A (2012) Anatomically-constrained tractography: improved diffusion MRI streamlines tractography through effective use of anatomical information. Neuroimage 62: 1924–1938. 10.1016/j.neuroimage.2012.06.005 22705374

[70] Mangin JF, Fillard P, Cointepas Y, Le Bihan D, Frouin V, Poupon C (2013) Toward global tractography. Neuroimage 80: 290–296. 10.1016/j.neuroimage.2013.04.009 23587688

[71] Sherbondy AJ, Dougherty RF, Napel S, Wandell BA (2008) Identifying the human optic radiation using diffusion imaging and fiber tractography. J Vis 8: 12 11–11. 10.1167/8.10.12 PMC2759943 19146354

[72] Benjamin CF, Singh JM, Prabhu SP, Warfield SK (2014) Optimization of tractography of the optic radiations. Hum Brain Mapp 35: 683–697. 10.1002/hbm.22204 23225566 PMC4083652

[73] Ogawa S, Takemura H, Horiguchi H, Terao M, Haji T, Pestilli F, et al (2014) White matter consequences of retinal receptor and ganglion cell damage. Invest Ophthalmol Vis Sci 55: 6976–6986. 10.1167/iovs.14-14737 25257055 PMC4215745

[74] Allen B, Spiegel DP, Thompson B, Pestilli F, Rokers B (2015) Altered white matter in early visual pathways of human amblyopes. Vision Res 114: 48–55. 10.1016/j.visres.2014.12.021 25615840

[75] Wakana S, Caprihan A, Panzenboeck MM, Fallon JH, Perry M, Gollub RL, et al (2007) Reproducibility of quantitative tractography methods applied to cerebral white matter. Neuroimage 36: 630–644. 17481925 10.1016/j.neuroimage.2007.02.049 PMC2350213

[76] Yeatman JD, Dougherty RF, Myall NJ, Wandell BA, Feldman HM (2012) Tract profiles of white matter properties: automating fiber-tract quantification. PLoS One 7: e49790 10.1371/journal.pone.0049790 23166771 PMC3498174

[77] Catani M, Thiebaut de Schotten M (2008) A diffusion tensor imaging tractography atlas for virtual in vivo dissections. Cortex 44: 1105–1132. 10.1016/j.cortex.2008.05.004 18619589

[78] Yendiki A, Panneck P, Srinivasan P, Stevens A, Zöllei L, Augustinack J, et al (2011) Automated probabilistic reconstruction of white-matter pathways in health and disease using an atlas of the underlying anatomy. Front Neuroinform 5: 23 10.3389/fninf.2011.00023 22016733 PMC3193073

[79] Catani M, Jones DK, Donato R, Ffytche DH (2003) Occipito-temporal connections in the human brain. Brain 126: 2093–2107. 12821517 10.1093/brain/awg203

[80] Oishi K, Huang H, Yoshioka T, Ying SH, Zee DS, Zilles K, et al (2011) Superficially located white matter structures commonly seen in the human and the macaque brain with diffusion tensor imaging. Brain Connect 1: 37–47. 10.1089/brain.2011.0005 22432953 PMC3569096

[81] Markov NT, Ercsey-Ravasz M, Van Essen DC, Knoblauch K, Toroczkai Z, Kennedy H (2013) Cortical high-density counterstream architectures. Science 342: 1238406. 10.1126/science.1238406 PMC3905047 24179228

[82] Sporns O (2013) The human connectome: origins and challenges. Neuroimage 80: 53–61. 10.1016/j.neuroimage.2013.03.023 23528922

[83] Reese TG, Heid O, Weisskoff RM, Wedeen VJ (2003) Reduction of eddy-current-induced distortion in diffusion MRI using a twice-refocused spin echo. Magn Reson Med 49: 177–182. 12509835 10.1002/mrm.10308

[84] Friston KJ, Ashburner J (2004) Generative and recognition models for neuroanatomy. Neuroimage 23: 21–24. 15325348 10.1016/j.neuroimage.2004.04.021

[85] Glasser MF, Sotiropoulos SN, Wilson JA, Coalson TS, Fischl B, Andersson JL, et al (2013) The minimal preprocessing pipelines for the Human Connectome Project. Neuroimage 80: 105–124. 10.1016/j.neuroimage.2013.04.127 23668970 PMC3720813

[86] Sotiropoulos SN, Jbabdi S, Xu J, Andersson JL, Moeller S, Auerbach EJ, et al (2013) Advances in diffusion MRI acquisition and processing in the Human Connectome Project. Neuroimage 80: 125–143. 10.1016/j.neuroimage.2013.05.057 23702418 PMC3720790

[87] Fischl B (2012) FreeSurfer. Neuroimage 62: 774–781. 10.1016/j.neuroimage.2012.01.021 22248573 PMC3685476

[88] Yushkevich PA, Piven J, Hazlett HC, Smith RG, Ho S, Gee JC, et al (2006) User-guided 3D active contour segmentation of anatomical structures: significantly improved efficiency and reliability. Neuroimage 31: 1116–1128. 16545965 10.1016/j.neuroimage.2006.01.015

[89] Tournier JD, Calamante F, Connelly A (2007) Robust determination of the fibre orientation distribution in diffusion MRI: non-negativity constrained super-resolved spherical deconvolution. Neuroimage 35: 1459–1472. 17379540 10.1016/j.neuroimage.2007.02.016

[90] Kim D, Sra S, Dhillon S (2013) A non-monotonic method for large-scale non-negative least squares. Optimization Methods and Software 28: 1012–1039.

[91] Kay KN, David SV, Prenger RJ, Hansen KA, Gallant JL (2008) Modeling low-frequency fluctuation and hemodynamic response timecourse in event-related fMRI. Hum Brain Mapp 29: 142–156. 17394212 10.1002/hbm.20379 PMC6871156

[92] Dumoulin SO, Wandell BA (2008) Population receptive field estimates in human visual cortex. Neuroimage 39: 647–660. 17977024 10.1016/j.neuroimage.2007.09.034 PMC3073038

[93] Wandell BA, Winawer J (2011) Imaging retinotopic maps in the human brain. Vision Res 51: 718–737. 10.1016/j.visres.2010.08.004 20692278 PMC3030662

