# Supplementary material for: Ensemble Tractography
Source: PLoS Comput Biol. 2016 Feb 4;12(2):e1004692. doi: 10.1371/journal.pcbi.1004692 (PMC4742469; doi:10.1371/journal.pcbi.1004692)
Supplement: S1 Text — (DOCX) [file pcbi.1004692.s001.docx]

**Supporting information of**

**Ensemble tractography**

Hiromasa Takemura^1,2,3,4^, Cesar F. Caiafa^5^, Brian A. Wandell^4^, & Franco Pestilli^6^

**Affiliation:**

^1^ Center for Information and Neural Networks (CiNet), National Institute of Information and Communications Technology, and Osaka University, Suita, Japan

^2^ The Japan Society for the Promotion of Science, Tokyo, Japan

^3^ Graduate School of Frontier Biosciences, Osaka University, Suita, Japan

^4^ Department of Psychology, Stanford University, CA, USA

^5^ Instituto Argentiono de Radioastronom[í](http://www.iar.unlp.edu.ar/index.html)a (IAR) - CCT La Plata - CONICET, Villa Elisa, Buenos Aires, Argentina

^6^ Department of Psychological and Brain Sciences, Indiana University, Bloomington, IN; Programs in Neuroscience and Cognitive Science; Indiana University Network Science Institute, Indiana University, Bloomington, IN USA.

**Contact information:**

Hiromasa Takemura

Center for Information and Neural Networks (CiNet), National Institute of Information and Communications Technology, and Osaka University

[htakemur@nict.go.jp](mailto:htakemur@nict.go.jp)

Franco Pestilli

Department of Psychological and Brain Sciences, Indiana University

[franpest@indiana.edu](mailto:franpest@indiana.edu)

**1. Testing streamline curvature distribution using PICo.**

To check the generality of streamline curvature distribution for different algorithms, we tested the probabilistic tractography implemented in Camino (PICo; Parker et al., 2003; <http://cmic.cs.ucl.ac.uk/camino/>). We generated whole-brain connectome using PICo with 4 different angle threshold settings (47.2, 23.1, 11.5 and 5.7 deg) while other parameters set default (step size = 0.2 mm, number of iteration on each seed voxel = 1). We used the STN96 dataset (see Materials and Methods). The subsequent streamline curvature analysis is identical to the analysis using MRtrix (see “Measuring mean streamline curvature” in “Materials and Methods” section in the main text).

# **2. Comparison between ETC and BigSPC model.**

We compared the ETC model accuracy with SPC 2 mm model accuracy. This was the best model among SPCs in occipital white matter (Fig. 5b). For the comparison, we matched candidate connectome sizes (“BigSPC model”). The flow diagram of BigSPC is shown in S6a Fig. Specifically, we generated 800,000 candidate streamlines using a single parameter (the minimum radius of curvature = 2 mm) and optimized the candidate connectome using LiFE. The accuracy of this BigSPC was compared with ETC model described in the main text (800,000 candidate streamlines derive from five different curvature threshold; 0.25, 0.5, 1, 2 and 4 mm). Analyses were performed using the occipital white matter in five individual brains from the STN96 dataset (see Materials and Methods; S2 Fig). Optimized ETC predicts diffusion signal better and covers wider range of white matter as compared with BigSPC (Figs. S6c-S6e). The ETC model is superior to any tested SPC with identical (large) number of candidate streamlines.

# **3. ET using multiple tractography algorithms.**

We tested how the ensemble tractography with candidate connectomes combined from multiple algorithms influences the accuracy and properties of connectome models.

We generated candidate connectomes by combining Single Algorithm Connectomes (SACs) derived from three different tractography algorithms that are implemented in MRtrix (DT_STREAM, tensor streamlines; SD_STREAM, CSD deterministic streamlines; SD_PROB, CSD probabilistic streamlines; Tournier et al., 2012). To test the effect of ensemble tractography across algorithms rather than parameter settings, we set consistent parameters across the three algorithms (step size: 0.2 mm; maximum length: 200 mm; minimum length: 10 mm; minimum radius of curvature: 1 mm). For CSD tractography, we set the maximum number of harmonics to 8 (*L_max_*= 8). Analyses were performed using the occipital white matter in five individual brains from the STN96 dataset (see Materials and Methods; S2 Fig).

We set the number of streamlines in the SAC candidate connectomes to be 120,000. We combine three candidate SACs to generate the candidate ETC (360,000 streamlines; S8a Fig). The optimized ETC contains more streamlines (S8b Fig), covers larger portion of white matter (S8c Fig) and predicts diffusion signal better than any of the SACs (S8d Fig).

# **4. ET using multiple stopping criterion parameters.**

We tested the improvement in accuracy of the ETC by sweeping over four different FOD amplitude stopping criterions in CSD-based probabilistic tractography on MRtrix (Tournier et al., 2012). We generated candidate ETC by combining SPCs derived from four different FOD amplitude stopping criteria (0, 0.05, 0.1 and 0.2) in the probabilistic tractography implemented in MRtrix, while other parameters are consistent (step size: 0.2 mm; maximum length: 200 mm; minimum length: 10 mm; minimum radius of curvature: 1 mm; vector specifying the initial direction: 20 deg; the maximum number of spherical harmonics: 8). Analyses were performed using the occipital white matter in five individual brains from the STN96 dataset (see Materials and Methods; S2 Fig).

We set the number of streamlines in the SPC candidate connectomes to be 160,000. We combined all streamlines from SPCs in order to generate ETC candidate (S9a Fig). Both SPCs and ETC are optimized using LiFE. The optimized ETC contains more streamlines (S9b Fig), covers larger portion of white matter (S9c Fig) and predicts diffusion signal better than any of the individual SPCs (S9d Fig).

# **5. Alternative ET approaches**

**ETC with preselection (ETCpre)**

In this alternative ET architecture, we select a subset of streamlines from each SPC, and combine those streamlines to create a new candidate ETC model.

We selected the streamlines as follows. We used LiFE to estimate the contribution of individual streamlines for predicting diffusion signal (streamline weight) in each SPC model. We then rank-ordered the streamlines in each SPC model based on their LiFE weight, and selected the top 32,000 with highest weight. Finally, we concatenated the top weighted streamlines from five SPC models to generate a new candidate ETC model (160,000 streamlines in total). S10a Fig describes the flowchart of the ETCpre. Analyses were performed using the occipital white matter in five individual brains from the STN96 dataset (see Materials and Methods; S2 Fig).

ETCpre was similar to the ETC built by including all SPC streamlines. The similarities were increased optimized connectome size (S10b Fig) and larger white matter coverage (S10c Fig), as compared with SPCs. The model accuracy of ETCpre was slightly worse than ETC, but significantly better than the SPCs (S10d Fig).

ETCpre has some advantage because it reducing the computational demand required by the increase in connectome model in whole-brain models. In fact, the candidate connectome of ETCpre maintains a size equal to each individual SPCs. We utilized the ETCpre architecture also to produce the whole-brain results presented in Fig. 7.

# **References**

Parker GJM, Haroon HA, Wheeler-Kingshott CAM (2003) A framework for a streamline-based probabilistic index of connectivity (PICo) using a structural interpretation of MRI diffusion measurements. Journal of Magnetic Resonance Imaging 18: 242-254.

Tournier JD, Calamante F, Connelly A (2012) MRtrix: Diffusion tractography in crossing fiber regions. International Journal of Imaging Systems and Technology 22: 53-66.
