# Supplementary figures and images for: Ensemble Tractography
Source: PLoS Comput Biol. 2016 Feb 4;12(2):e1004692. doi: 10.1371/journal.pcbi.1004692 (PMC4742469; doi:10.1371/journal.pcbi.1004692)

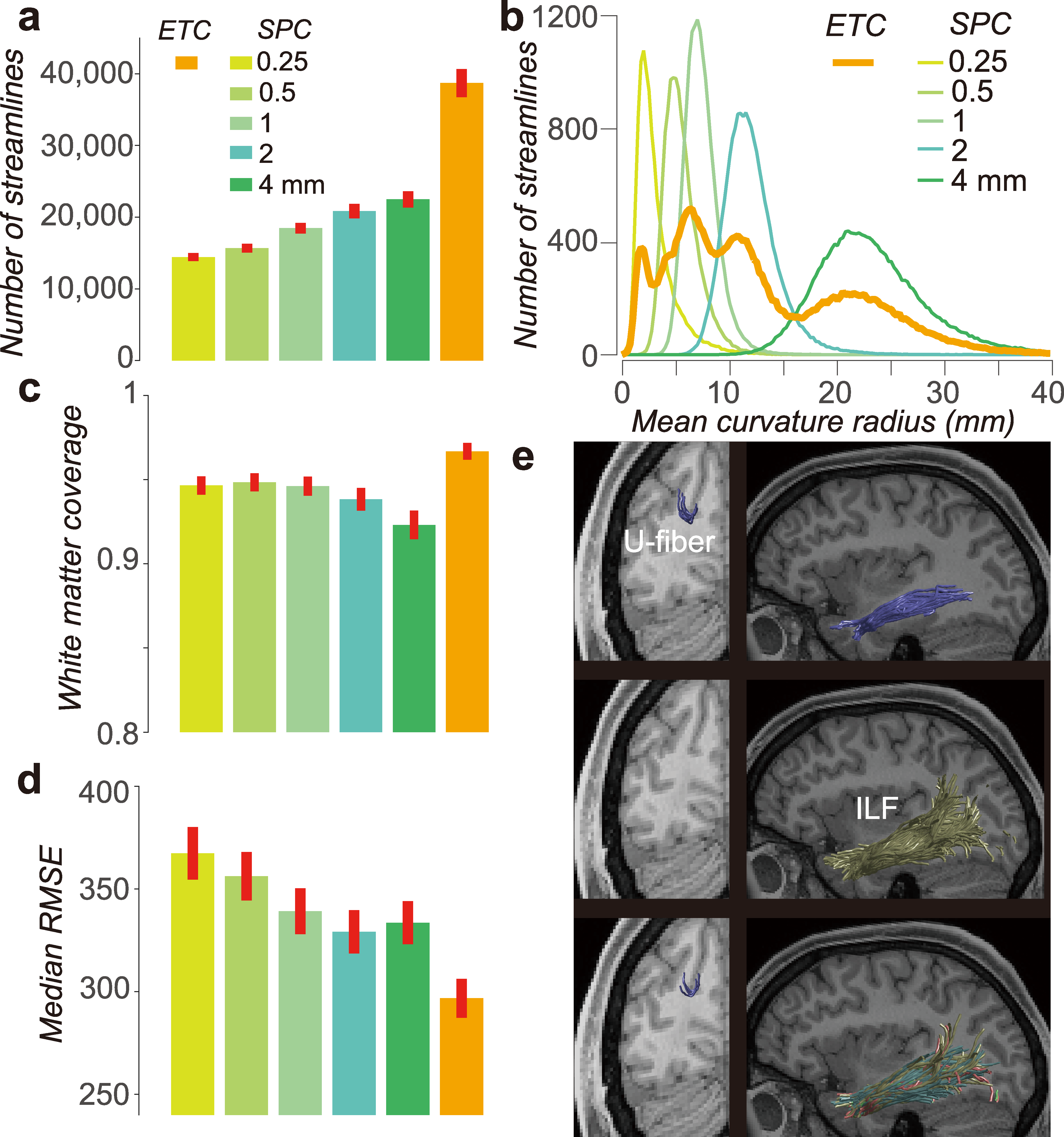

Supplement: S7 Fig — a. Optimal connectome size in 8 hemispheres from HCP90 dataset (occipital cortex). Error bar depicts ±1 s.e.m. across hemispheres. Conventions are identical to those in Fig 4a and 4b. Distribution of radius of curvature in optimized connectome in six connectome models (SPCs and ETC), averaged across 8 hemispheres. Conventions are identical to those in Fig 3. c. White matter coverage. d. Comparison of Root Mean Squared Error (RMSE) between measured and predicted diffusion signal across connectome models. Other conventions are identical to those in Fig 5b. e. U-fiber and ILF supported by different connectome models. In SPC model using 2mm, there are no streamlines in the optimized connectome projecting two gyri in dorsal visual cortex. Conventions are identical to Fig 1. (TIF) [file pcbi.1004692.s008.tif]
